# Pulmonary Hypertension in Pregnancy: Critical Care Management

**DOI:** 10.1155/2012/709407

**Published:** 2012-07-05

**Authors:** Adel M. Bassily-Marcus, Carol Yuan, John Oropello, Anthony Manasia, Roopa Kohli-Seth, Ernest Benjamin

**Affiliations:** Division of Critical Care Medicine, Department of Surgery, Mount Sinai School of Medicine, P.O. BOX 1264, New York, NY 10029, USA

## Abstract

Pulmonary hypertension is common in critical care settings and in presence of right ventricular failure is challenging to manage. Pulmonary hypertension in pregnant patients carries a high mortality rates between 30–56%. In the past decade, new treatments for pulmonary hypertension have emerged. Their application in pregnant women with pulmonary hypertension may hold promise in reducing morbidity and mortality. Signs and symptoms of pulmonary hypertension are nonspecific in pregnant women. Imaging workup may have undesirable radiation exposure. Pulmonary artery catheter remains the gold standard for diagnosing pulmonary hypertension, although its use in the intensive care unit for other conditions has slowly fallen out of favor. Goal-directed bedside echocardiogram and lung ultrasonography provide attractive alternatives. Basic principles of managing pulmonary hypertension with right ventricular failure are maintaining right ventricular function and reducing pulmonary vascular resistance. Fluid resuscitation and various vasopressors are used with caution. Pulmonary-hypertension-targeted therapies have been utilized in pregnant women with understanding of their safety profile. Mainstay therapy for pulmonary embolism is anticoagulation, and the treatment for amniotic fluid embolism remains supportive care. Multidisciplinary team approach is crucial to achieving successful outcomes in these difficult cases.

## 1. Introduction

Pregnancy in women with pulmonary hypertension is known to be associated with significantly high mortality rate between 30% and 56% [1]. The physiologic changes that occur during pregnancy and the peripartum period are poorly tolerated in these patients. There are also acute conditions associated with pregnancy that may be complicated by severe pulmonary hypertension, such as, pulmonary and amniotic fluid embolism. Majority of maternal deaths occur during labor or within 1 month postpartum [2].

Pulmonary hypertension is defined as an increase in mean pulmonary artery pressure (PAP) (mPAP) >25 mmHg at rest as assessed by right heart catheterization (RHC). Recent developments have been made in the treatment of pulmonary hypertension, and advances in the multidisciplinary approach are believed to have an impact on the high maternal mortality rate [3]. However, management of critically ill patients with hemodynamically significant pulmonary hypertension remains challenging. In this paper we review the diagnosis and treatment of critically ill parturient with pulmonary hypertension of different etiologies and discuss treatment strategies.

## 2. Pregnancy and Labor Physiology

**Figure 1:**
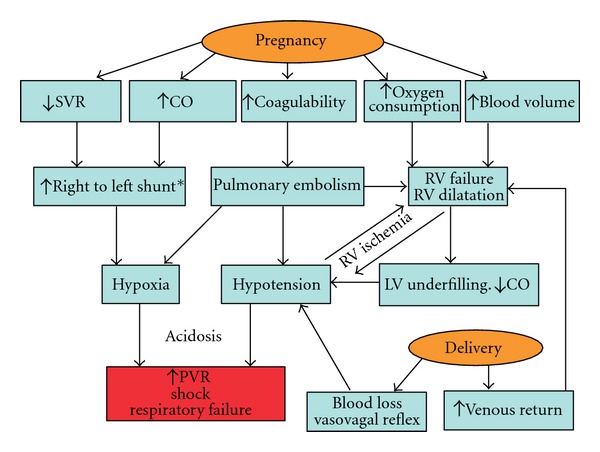
The physiologic response to pregnancy in pulmonary arterial hypertention* = right to left shunt increases in Eisenmenger's patients and patients with a patent foramen ovale [7].

During pregnancy, several physiologic changes further impact on the hemodynamic ramifications in pulmonary hypertension [PH] (Figure 1). Virtually every organ system is affected in pregnancy. The most significant change in the cardiovascular system is increase in blood volume, which can increase in a normal, healthy pregnant female almost 50% above the nonpregnant level at it peaks during 20–32 weeks of gestation [4]. In addition, heart rate and stroke volume are also increased with higher cardiac output. Systemic and pulmonary vascular resistances (PVRs) are decreased. However, in women with pulmonary hypertension, pulmonary vascular disease prevents the fall in PVR, leading to further rise in PAP with increased cardiac output [5]. Due to the stimulation of progesterone, tidal volume is increased despite the elevation of the diaphragm, whereas respiratory rate is unchanged. The rise in tidal volume accounts for increased minute volume and respiratory alkalosis with a mean arterial partial carbon dioxide pressure (PCO_2_) of 30 mmHg and a decreased functional residual capacity [6].

Labor and delivery feature a further increase in cardiac output and blood pressure particularly during uterine contractions. These hemodynamic modifications are heavily influenced by the mode of delivery. Normal vaginal delivery is associated with a 34% increase in cardiac output at full cervical dilation [4]. At the point of cesarean section delivery and in response to spinal anesthesia, a 47% increase in cardiac index and 39% decrease in SVR have been recorded [6, 8]. Following delivery, several factors lead to hemodynamic instability in the PH patients, including decreased preload from blood loss and anesthesia, increased preload from relief of caval obstruction, or additional blood return from the contracting uterus, abrupt increase of SVR and PVR to nonpregnancy state, and reduced ventricular contractility [2, 4, 9].

A normal pregnancy induces a hypercoagulable state due to a combination of physical and hormonal factors, as well as hematologic changes. Progesterone-mediated increases in venous distensibility and capacity lead to increased venous stasis. The enlarging uterus may also induce a selective compressive effect on the common iliac vein. Pregnancy causes hematologic changes including increased circulating levels of clotting factors, decreased protein S levels and resistance to activated protein C [10]. The generation of fibrin is increased, and fibrinolytic activity is decreased. The combination of these factors results in a hypercoagulable state.

## 3. Pathophysiology

Multiple molecular pathways have been implicated in the pathogenesis of pulmonary hypertension. Vaso-affective molecules produced in the pulmonary vascular endothelium include nitric oxide and prostacyclin, which are vasodilators. Endothelin-1 acts as a vasoconstrictor and is involved in vascular smooth muscle proliferation [11]. Thus, dysfunction of the molecular pathways and dysregulation of their production can lead to imbalance between vasodilation and vasoconstriction and between apoptosis and proliferation. These molecular alternations are thought to be the underlying disease mechanisms for chronic pulmonary arterial hypertension [12].

In acute pulmonary hypertension, hypoxic pulmonary vasoconstriction plays an important role and can be the inciting or perpetuating factor for increased pulmonary pressures. In acute lung injury (ALI)/acute respiratory distress syndrome (ARDS), both hypoxic vasoconstriction and deposition of intravascular fibrin and cellular debris contribute to vascular obliteration and PH [13]. Endotoxin release in sepsis has been shown in animal studies to cause PH by causing constriction of proximal pulmonary arteries and decreased compliance of the distal pulmonary vasculature [12]. In massive acute pulmonary embolism, the increase in pulmonary vascular resistance is related to the mechanical obstruction from the thrombosis load and subsequent vasoconstriction [14]. Vascular obstruction was historically thought to be also the pathophysiology in amniotic fluid embolism (AFE). However, more recent evidence suggests pulmonary hypertension is due to vasoactive substances (prostacyclin, endothelin) or immunologic factors. The latter supported by decreased complement level measured in postpartum AFE patients compared to control. These findings indicate AFE may result from biochemical mediators released after the embolization occurs and have led some authors to propose renaming the entity “anaphylactoid syndrome of pregnancy” [15].

Pulmonary hypertension of different causes can lead to a final common pathway of right ventricular strain or failure. Right ventricle is a thin walled structure that tolerates poorly acute increase in afterload. This is because right ventricular stroke volume decreases proportionately to acute increase in afterload, and it cannot acutely increase the mean PAP to more than 40 mmHg [11]. The RV subsequently becomes dilated, which is in contrast to chronic pulmonary hypertension, where RV hypertrophy is the main feature reflecting an adaptive mechanism. RV distention in turn results in increase oxygen consumption and reduction in contractility. It could also impair left ventricular (LV) filling with paradoxic interventricular septal movement, leading to decreased cardiac output and oxygen delivery [16]. Perfusion of the right coronary artery is usually dependent on adequate aortic root pressure and a pressure gradient between the aorta and RV [17]. In setting of increased RV pressure and decreased cardiac output, RV ischemia may ensue, with further severe hemodynamic decompensation.

## 4. Classification and Etiologies of PH

The world health organization (WHO) classification of pulmonary hypertension has been redefined and updated in 2009 (Table 1) [18]. Idiopathic PAH and PAH associated with connective tissue disease affect predominantly women of childbearing age [18]. Idiopathic PAH is rare and a rapidly progressive disease with an untreated survival of only 2.8 years [19]. The association of PAH with connective tissue disease is a common phenomenon. The highest incidence of the development of PAH is known in scleroderma patients, especially with the CREST syndrome (10–20% develop PAH) followed by systemic lupus erythematous (SLE, 10%) [20]. Patients with PAH in connective tissue disease have a deleterious clinical course and a worse prognosis [18].

WHO group 1 also includes PAH associated with congenital heart disease (CHD). The disease could be further classified based on anatomic pathophysiology of shunts or clinical phenotypes (Tables 2 and 3). A significant proportion of patients with CHD, in particular those with relevant systemic-to-pulmonary shunts, will develop PAH if left untreated [18]. Hemodynamic changes during pregnancy can exacerbate the problems associated with CHD as well. In the Eisenmenger syndrome, right to left shunting increases during pregnancy because of systemic vasodilation and RV overload with decrease in pulmonary blood flow and increase cyanosis. The outcome is related to functional class (NYHA classification), the nature of the disease, and previous cardiac surgery. Any patient in functional class III or IV during pregnancy is at high risk whatever the underlying condition as this means there is no remaining cardiovascular reserve [5]. The high-risk conditions are fragile aortas as in Marfan syndrome, left sided obstructions, and already dilated poorly functioning left ventricles [9].

Pregnancy is often fatal for a PAH patient. In a retrospective review study from 1978 to 1996, mortality was 30% in IPAH, 36% in Eisenmenger's syndrome, and 56% in PH secondary to other conditions [1]. A systemic review of all published reports from 1997 to 2007 of pregnancies in women with PAH found that overall maternal mortality was lower than previous reports, thought may be attributable to use of targeted PAH therapies and improved understanding of the disease. Mortality was 17% in IPAH, 28% in PAH associated with congenital heart disease, and 33% in PAH of other etiologies [21]. A recent prospective, multinational registry that included 26 pregnancies reported improved mortality in well-controlled and particularly long-term responders to calcium channel blockers [22]. Venous thromboembolism affects pregnant women 5 times more frequently than nonpregnant women of similar age [10]. Pulmonary embolism has surpassed infection, hemorrhage, and preeclampsia/eclampsia to become a leading cause of manternal mortality in the United States [10]. Amniotic fluid embolism (AFE) is a rare but catastrophic complication unique to pregnancy. Despite variation in reported incidence and mortality, AFE remains a life-threatening condition with significant morbidity and mortality for the pregnant women [15, 23–25]. It is the 5th most common cause of maternal mortality in the world [15].

## 5. Diagnosis and Evaluation

### 5.1. History and Physical Examination

Symptoms of pulmonary hypertension include chest pain, cough, and shortness of breath. With right heart failure patients may also have lower extremity swelling, dizziness, or syncope. Many of these symptoms overlap with that of normal pregnancy. Physical examination of patients with pulmonary hypertension and right ventricular failure reveals a prominent pulmonic component of the second heart sound and an elevated jugular venous pulse. Other findings may include a palpable right ventricular heave and systolic murmur of tricuspid regurgitation along the left lower sternal border. Accentuation of this murmur during inspiration (Carvallo's sign) distinguishes it from the murmurs of mitral regurgitation and aortic stenosis [11]. The lung examination may suggest underlying lung disease. Patients with isolated right ventricular failure do not have pulmonary edema, which if found, suggests left ventricular dysfunction, pulmonary venous hypertension, or ARDS.

### 5.2. Blood Tests

Biomarkers, such as, brain natriuretic peptide (BNP) are useful in monitoring chronic PAH [26]. In pulmonary embolism, BNP can stratify patients regarding risk for development of right ventricular failure [27] and troponin I leak may predict mortality [28]. Measurement of renal, liver, and neurological function will provide some information about the adequacy of cardiac function and tissue perfusion.

### 5.3. Chest X-Ray

Plain chest radiography (CXR) is of limited utility in diagnosing pulmonary hypertension in the ICU. Typical findings of right ventricular hypertrophy, right atrial enlargement, and obscuring of the aortopulmonary window by enlarged pulmonary arteries are less obvious on portable radiographs. Nonetheless, diffuse severe pulmonary parenchymal abnormalities may suggest an underlying cause of pulmonary hypertension. In pregnant women with suspected pulmonary embolism, CXR is recommended as the first radiation-associated imaging work-up; lung scintigraphy as the preferred test in the setting of a normal CXR, followed by computed-tomographic pulmonary angiography (CTPA) if the ventilation-perfusion result is nondiagnostic [29].

### 5.4. Right Heart Catheterization

Right heart or pulmonary artery (PA) catheterization is the gold standard for the diagnosis of pulmonary hypertension. Its use has fallen out of favor in the critically ill patients in general due to lack of studies with positive outcomes. However, there have been no studies targeting specifically the “pulmonary vascular” subpopulation. Most authors recommend the placement of PA catheters for patients admitted to the ICU with severe PH and RV failure, allowing for continuous measurement of RA and pulmonary pressures, cardiac output and mixed venous oxygen saturation [11, 14, 16, 30]. Certain technical and interpretive limitations should be recognized. Severe tricuspid regurgitation and elevated PAP can make catheter placement challenging. Determination of cardiac output by thermodilution may be erroneously low because of tricuspid regurgitation. The Fick's method may be more accurate but requires determination of oxygen consumption, which is challenging in critically ill patients. PVR is a composite index of pulmonary pressure and cardiac output. However, PVR may not accurately reflect right ventricular afterload. Ultimately the response to a treatment strategy should be guided by the adequacy of tissue oxygen, which is partly reflected by central venous oxygen saturations.

### 5.5. Ultrasound

Compression ultrasound (CUS) is a noninvasive test with sensitivity of 97% and a specificity of 94% for the diagnosis of symptomatic, proximal deep venous thrombosis (DVT) in the general population [10]. CUS does not involve any radiation exposure and is recommended for evaluation of venous thromboembolism in pregnant women with signs and symptoms of DVT, with or without suspected PE [29]. Goal-directed bedside ultrasound has gained recognition in critical care. It can also be applied to the evaluation of patients with pulmonary hypertension. Echocardiography provides direct and noninvasive visualization of the right ventricle allowing intermittent repetitive followup of the dynamics of therapeutic responses. A recent statement of the American College of Chest Physicians (ACCPs) and the French Society of Intensive Care Medicine (SRLF) reported that a simple evaluation of the right ventricle can be done by nonexpert physicians or a more sophisticated evaluation by experts [31]. Right ventricular systolic pressure can be estimated from the tricuspid regurgitation velocity, assuming no significant right ventricular outflow tract obstruction [32]. The most usual echocardiographic sign of RV dilatation is the loss of its typical triangular shape. Right ventricular size can also be assessed by calculating the RV/LV end-diastolic area ratio in the four-chamber view. A normal ratio is below 0.6. When the RV is larger than the LV, the RV is defined as severely dilated. Another specific sign of RV failure is the paradoxical septal motion in systole with shifting to the left ventricle (D shaped septum), reflecting RV overload [16]. Lung ultrasonography may be integrated into bedside evaluation as an adjunct to the standard chest radiograph and CT scan. Normally aerated lung shows an A-line pattern, which is a reverberation artifact. The presence of A-line pattern indicates that the pulmonary artery occlusion pressure is <18 mm Hg and rules out cardiogenic pulmonary edema. B lines indicate an abnormality in the interstitial or alveolar compartment. These are comet-tail artifacts project from the pleural line, move with respiration, and extend to the bottom of the ultrasound screen. Diffuse B-line pattern may result from cardiogenic pulmonary edema and is associated with a smooth pleural line. B lines resulting from noncardiogenic lung injury, such as, ARDS, or interstitial lung disease, are associated with an irregular pleural surface and a nonhomogeneous B-line distribution with small subpleural areas of lung consolidation [33]. Lung ultrasonography may help differentiate the causes of pulmonary hypertension in critical care and minimizes radiation exposure in pregnant patients.

## 6. General Management in the Intensive Care Unit

 Improved survival in pregnancy and pulmonary hypertension in recent years is attributable in addition to the new treatment modalities, incorporation of a multidisciplinary approach is equally important [2, 20, 21, 34]. Pulmonary hypertension in pregnancy and critical care is a complex clinical entity that requires collaborative efforts between obstetricians, anesthesiologists, cardiologists, pulmonologists, and intensivists. There is no standardized approach to the management of PH in pregnancy, successful outcomes are heavily dependent on a methodical approach individualized to each patient developed by a multidisciplinary team in a dedicated intensive care unit.

### 6.1. Fluid Management

Fluid management of these patients is often difficult, as both hypovolemia and hypervolemia can have detrimental effects. Unmonitored fluid challenges may further impair RV function and are not recommended. RV volume overload may be identified by a rising V wave on the central venous pressure (CVP) trace or by increased tricuspid regurgitation seen on echocardiography. In the situation with predominantly diastolic RV dysfunction, management involves fluid removal by diuresis or hemofiltration.

### 6.2. Inotropic Augmentation of RV Myocardial Function

Systolic RV failure with low cardiac output and hypotension may require inotropic agents. For sympathomimetic agents, desirable cardiac *β*_1_ effects may be offset by chronotropic effects precipitating tachyarrhythmias, as well as worsening pulmonary vasoconstriction at higher doses through-agonism. Dobutamine has favorable pulmonary vascular effects at lower doses, although it leads to increased PVR, tachycardia, and systemic hypotension at doses exceeding 10 mcg/kg/min. Dopamine may increase tachyarrhythmias and is not recommended in the setting of cardiogenic shock. Alternatively, agents that do not have chronotropic properties, such as, phosphodiesterase (PDE)-3 inhibitors, may be preferable. PDE-3 usually deactivates intracellular cyclic adenosine monophosphate (cAMP), and PDE-3 inhibitors therefore increase cAMP and augment myocardial contractility while dilating the vasculature. Milrinone is frequently used, and nebulized milrinone, through pulmonary selectivity, has less systemic hypotension and V/Q mismatch compared with intravenous use [16]. Levosimendan, a calcium-sensitizing agent with positive inotropic and vasodilatory effects, holds promise for patients with PH and RV failure, but it has not yet been thoroughly investigated in these patients [17].

### 6.3. Vasopressors

An essential goal of using vasopressor is to maintain systemic blood pressure above pulmonary arterial pressures, thereby preserving right coronary blood flow and preventing shunt. Vasopressors will, however, inevitably have direct effects on the pulmonary circulation as well as myocardial effects. Norepinephrine improves RV function both by improving SVR and CO although may increase PVR at higher doses. Phenylephrine is a direct alpha agonist, it improves right coronary perfusion in RV failure without causing tachycardia, although this benefit may be offset by worsening RV function due to increased PVR. Arginine vasopressin (AVP) causes systemic vasoconstriction via the vasopressinergic (V1) receptor. At low dose it has demonstrated vasodilating properties that manifest clinically as a reduction in PVR and PVR/SVR ratio. Low-dose AVP may be useful in difficult cases with vasodilatory shock and pulmonary hypertension, but further investigation is required [11].

The pregnant uterus has both *α* and *β* adrenergic receptors. The *α* receptor activation causes an increase in uterine muscle tone, whereas *β* receptor stimulation causes a decrease in uterine muscle activity. The vasculature of the uterus has only *α* receptors, so while *β* stimulating agents do not affect uterine blood flow, *α* receptor activators can cause uterine vasoconstriction with a decrease in blood flow [35].

### 6.4. Pulmonary Vasodilators

One of the important interventions to reverse RV failure is to reduce RV afterload through the use of pulmonary vasodilators. PAH-targeted therapies have revolutionized the care of patients with PAH. Agents are classically subdivided according to their action on the cyclic GMP, prostacyclin, or endothelin pathways. Endothelin receptor antagonists are pregnancy category X drugs and are contraindicated in pregnancy. These agents have been associated with profound craniofacial, cardiovascular, and visceral malformations in the rat model [2]. Calcium channel blockers are recommended for “responders” to vasodilator testing [36]. Their prolonged half life and negative inotropic effects, however, limit their use in treatment of acute pulmonary hypertension [30]. In addition, nifedipine, amlodipine, and diltiazem are all pregnancy category C drugs.

### 6.5. Prostaglandins

There have been only case reports describing successful use of targeted pulmonary vasodilator therapy. In pregnant patients presenting with PH and RV failure, intravenous epoprostenol is the initial treatment of choice [2, 7, 37–39], although care must be taken to avoid systemic hypotension. Both epoprostenol and treprostinil are classified as pregnancy category B. Most of the published case reports describe initiating intravenous epoprostenol several weeks before or near the time of delivery in parturients with PH [2, 7, 37, 38]. However, IV epoprostenol may inhibit platelet aggregation, so bleeding should be monitored particularly during delivery and postpartum period [40]. Nebulized Iloprost (category C) has also been used with positive outcomes although with more limited evidence [34, 39, 40].

### 6.6. PDE-5 Inhibitors

Sildenafil causes vasodilation of the pulmonary vascular bed and in the systemic circulation. It also has a positive inotropic effect on the hypertrophic right ventricle. It is a category B medication. Using sildenafil to treat PH in pregnancy has been described in case reports and appears to be safe, but experience is still limited [7, 39, 40].

### 6.7. Inhaled NO

Inhaled nitric oxide is a direct pulmonary vasodilator. It has been shown to have a beneficial effect on outcome of postoperative critically ill patients with severe pulmonary hypertension and/or right ventricular failure [17]. However, prolonged administration is associated with several adverse effects, such as, rebound pulmonary hypertension after withdrawal, production of NO_2_, and development of methemoglobinemia [14].

### 6.8. Preventing Thromboembolic Events

The practice of thromboprophylaxis or anticoagulation in pregnant women with PH is not standardized. Most case reports of pregnant patients with PH place patients on thromboprophylaxis during pregnancy and continue through postpartum, with only brief interruption around time of delivery [2, 3, 21, 34]. Exceptions are for those who have idiopathic PAH and history of thromboembolic diseases where higher levels of anticoagulation may be required, and in patients with PAH associated with congenital heart disease, where caution should be exercised if prior history of bleeding exists [21, 34, 40].

### 6.9. Delivery and Anesthesia

Mode of delivery and anesthetic management remain debated. Vaginal delivery may be preferred over cesarean section to minimize postsurgical fluid shifts [2] or increased anesthetic risks [5]. Cesarean section, on the other hand, provides for a more controlled setting, avoids a prolonged second stage of labor [5], the potential for uncontrolled vaginal hemorrhage, and the adverse hemodynamic effects of bearing down [3]. If vaginal delivery is used, it should be performed in the ICU or the operating room [7]. Delivery in the lateral position avoids fetal compression of the inferior vena cava. The goals of anesthetic management are to avoid pain, hypoxemia, hypercapnia, and acidosis; all of which lead to increased PVR and thus hypertension [20]. Spinal and general anesthesia causes peripheral vasodilatation and may worsen the patient's hemodynamic. Regional anesthesia may be advantageous, however, when used in large dosages, may produce a decrease in venous return because of a sympathetic block [3, 7]. Investigators have reported using combined spinal-epidural anesthesia to provide a better sensory block than epidural anesthesia alone and no additional risk of hypotension with the use of very low-dose spinal anesthesia [3, 6, 7].

## 7. Management for Specific Diseases Causing Acute PH

**(A) Pulmonary Embolism**

The mainstay of therapy for acute venous thromboembolic disease during pregnancy is heparin [10], which does not cross the placenta, so does not carry risks of fetal hemorrhage or teratogenesis. Low molecular weight heparin (LMWH) also does not cross the placenta and therefore may be safe during pregnancy. However, they may require monitoring of anti-Xa levels and frequent dose adjustments, negating their logistic benefits over unfractionated heparin. Coumadin derivatives cross the placenta and are associated with warfarin embryopathy and can cause fetal hemorrhage.

In massive pulmonary embolism (MPE), anticoagulation alone may be insufficient. Hemodynamic instability and right-heart strain may ensue. Subsequent treatment options, with variable level of evidence supporting their uses, include thrombolytics, embolectomy, and IVC filter. IVC filters have been successfully used during pregnancy, and the indications for their use are the same as for the nonpregnant population. These include recurrent embolism on adequate medical therapy, strong contraindications to full anticoagulation after a thromboembolism, and critically ill patients at high risk for recurrent embolism, in whom recurrent embolism is likely to be fatal [10]. IVC filters have been associated with a small but real risk of complications, especially over the long term. Risks include migration of the filter, perforation of the aorta, duodenum, or renal pelvis, and penetration of nearby structures including the vertebrae and the retroperitoneum [41]. For these reasons, retrievable filters can be an attractive alternative in this patient population, who are likely to be young and are at higher risk of long-term complications from indwelling filters.

The evidence on thrombolytic therapy in pregnant patients is limited to case reports [41, 42], and in fact, pregnancy is considered a relative contraindication for thrombolytic therapy. Systemic thrombolytic has high risk of major bleeding; however, some of the pregnancy-specific complications have not been reported, and it is not clear whether they are caused by the underlying disease or treatment. The risks and benefits of thrombolytics for MPE in pregnancy should be considered carefully on an individual basis. Data from the nonpregnant population indicate that thrombolytics can be considered for the treatment of patients who are hemodynamically unstable [43]. Recombinant tissue plasminogen activator and streptokinase do not cross the placenta, and their use is recommended if thrombolytic therapy is employed. Urokinase is a small molecule purified from human urine and crosses the placenta. It is not currently known whether urokinase induces a fetal coagulopathy [10]. Catheter-directed thrombolytic therapy carries the theoretical advantages of more rapid clot lysis and a lower risk of bleeding, because of a higher local concentration drug; however, there is no convincing evidence proving its superiority over systemic therapy [44]. On the other hand, disadvantages are radiation exposure associated with fluoroscopy. More experience is needed before catheter-directed therapy can be recommended for pregnant patients.

In a review on recent findings on management of PH, embolectomy and cardiopulmonary bypass was associated with a higher rate of fetal loss compared to thrombolytic therapy [14]. Although these data must be interpreted carefully, as they are limited to case studies and case reports, it suggests that embolectomy must be restricted to cases in which the life of the woman is endangered.

**(B) Amniotic Fluid Embolism**

The management of amniotic fluid embolism is supportive and focuses initially on rapid maternal cardiopulmonary stabilization [23]. The most important goal of therapy is to prevent additional hypoxia and subsequent end-organ failure. Supportive treatment modalities directed towards the maintenance of oxygenation, circulatory support with fluid resuscitation, vasopressors, and/or inotropes, and correction of the coagulopathy provide the basis for care. Several newer therapies for AFE have been described in case reports. Cardiopulmonary bypass, extracorporeal membrane oxygenation, and intra-aortic balloon counterpulsation have been used successfully [45] Right ventricular assist device (RVAD) has been described in successful management of AFE with severe pulmonary hypertension and RV failure [24]. Fetal delivery, if not yet occurred at time of diagnosis, should be performed immediately to prevent further hypoxic damage to the fetus and to facilitate cardiopulmonary resuscitative efforts [23].

## 8. Conclusion

In conclusion, PH in pregnancy carries a high mortality. The management of these patients in the ICU is challenging with unique pregnancy-related physiologic changes and concern for fetal safety. During the past decade, new advanced therapies for pulmonary hypertension and cardiopulmonary support devices have emerged. Their application in pregnant women is based on limited evidence and data extrapolated from the nonpregnant population. Improved maternal and fetal survival in recent years is attributable to improved understanding of pulmonary hypertension, advanced therapies, and adoption of a multidisciplinary treatment approach.

## Figures and Tables

**Table 1:** Venice clinical classification of pulmonary hypertension (2003).

(1) Pulmonary arterial hypertension (PAH)	
(1.1) Idiopathic (IPAH)	
(1.2) Familial (FPAH)	
(1.3) Associated with (APAH)	
(1.3.1) Collagen vascular disease	
(1.3.2) Congenital systemic-to-pulmonary shunts	
(1.3.3) Portal hypertension	
(1.3.4) HIV infection	
(1.3.5) Drugs and toxins	
(1.3.6) Other (thyroid disorders, glycogen storage disease,	
Gaucher disease, hereditary hemorrhagic	
telangiectasia, hemoglobinopathies,	
myeloproliferative disorders, splenectomy)	
(1.4) Associated with venous or capillary involvement	
(1.4.1) Pulmonary venoocclusive disease (PVOD)	
(1.4.2) Pulmonary capillary hemangiomatosis (PCH)	
(1.5) Persistent pulmonary hypertension of the newborn	
(2) Pulmonary hypertension with left-heart disease	
(2.1) Left-sided atrial or ventricular heart disease	
(2.2) Left-sided valvular heart disease	
(3) Pulmonary hypertension associated with lung diseases and/or	
hypoxemia	
(3.1) Chronic obstructive pulmonary disease	
(3.2) Interstitial lung disease	
(3.3) Sleep-disordered breathing	
(3.4) Alveolar hypoventilation disorders	
(3.5) Chronic exposure to high altitude	
(3.6) Developmental abnormalities	
(4) Pulmonary hypertension owing to chronic thrombotic and/or	
embolic disease	
(4.1) Thromboembolic obstruction of proximal pulmonary	
arteries	
(4.2) Thromboembolic obstruction of distal pulmonary	
arteries	
(4.3) Nonthrombotic pulmonary embolism (tumor, parasites,	
foreign material)	
(5) Miscellaneous	
Sarcoidosis, histiocytosis X, lymphangiomatosis, compression	
of pulmonary vessels (adenopathy, tumor, fibrosing	
mediastinitis)	

**Table 2:** Anatomic-pathophysiologic classification of congenital systemic-to-pulmonary shunts associated with pulmonary arterial hypertension.

(1) Type	
(1.1) Simple pretricuspid shunts	
(1.2) Simple posttricuspid shunts	
(1.3) Combined shunts	
(1.4) Complex congenital heart disease	
(2) Dimension (specify for each defect if >1 congenital heart	
defect)	
(2.1) Hemodynamic (specify Qp/Qs)^∗^: restrictive or	
nonrestrictive	
(2.2) Anatomic defect size: small, moderate or large	
(3) Direction of shunt	
(3.1) Predominantly systemic-to-pulmonary	
(3.2) Predominantly pulmonary-to-systemic	
(3.3) Bidirectional	
(4) Associated cardiac and extracardiac abnormalities	
(5) Repair status	
(5.1) Unoperated	
(5.2) Palliated	
(5.3) Repaired	

**Table 3:** Clinical classification of congenital systemic-to-pulmonary shunts associated with PAH.

*A. Eisenmenger syndrome*	
Includes all systemic-to-pulmonary shunts resulting from large defects that lead to severely increased PVR and a reversed or bidirectional shunt: multiple-organ involvement are present	
*B. PAH associated with systemic-to-pulmonary shunts*	
Includes moderate to large defects: PVR is mildly to moderately increased, systemic-to-pulmonary shunt is still prevalent, no cyanosis at rest	
*C. PAH with small defects*	
Small defects (usually VSD <1 cm and ASD <2 cm): clinical picture is similar to idiopathic PAH	
*D. PAH after corrective cardiac surgery*	
Congenital heart disease has been corrected, but PAH is still present without significant postoperative residual lesions	

PAH: pulmonary arterial hypertension; PVR: pulmonary vascular resistance; VSD: ventricular septal defect; ASD: atrial septal defect.
